# Whole Cell Screen for Inhibitors of pH Homeostasis in *Mycobacterium tuberculosis*

**DOI:** 10.1371/journal.pone.0068942

**Published:** 2013-07-30

**Authors:** Crystal M. Darby, Helgi I. Ingólfsson, Xiuju Jiang, Chun Shen, Mingna Sun, Nan Zhao, Kristin Burns, Gang Liu, Sabine Ehrt, J. David Warren, Olaf S. Anderson, Steven J. Brickner, Carl Nathan

**Affiliations:** 1 Department of Microbiology and Immunology, Weill Cornell Medical College, New York, New York, United States of America; 2 Department of Physiology and Biophysics, Weill Cornell Medical College, New York, New York, United States of America; 3 Milstein Chemistry Core Facility, Weill Cornell Medical College, New York, New York, United States of America; 4 Department of Biochemistry, Weill Cornell Medical College, New York, New York, United States of America; 5 Institute of Materia Medica, Chinese Academy of Medical Sciences & Peking Union Medical College, Beijing, P. R. China; 6 Tsinghua-Peking Center for Life Sciences and Department of Pharmacology and Pharmaceutical Sciences, School of Medicine, Tsinghua University, Beijing, P. R. China; 7 SJ Brickner Consulting, LLC, Ledyard, Connecticut, United States of America; University of Delhi, India

## Abstract

Bacterial pathogens like *Mycobacterium tuberculosis* (*Mtb*) encounter acidic microenvironments in the host and must maintain their acid-base homeostasis to survive. A genetic screen identified two *Mtb* strains that cannot control intrabacterial pH (pH_IB_) in an acidic environment; infection with either strain led to severe attenuation in mice. To search for additional proteins that *Mtb* requires to survive at low pH, we introduced a whole-cell screen for compounds that disrupt pH_IB_, along with counter-screens that identify ionophores and membrane perturbors. Application of these methods to a natural product library identified four compounds of interest, one of which may inhibit novel pathway(s). This approach yields compounds that may lead to the identification of pathways that allow *Mtb* to survive in acidic environments, a setting in which *Mtb* is resistant to most of the drugs currently used to treat tuberculosis.

## Introduction

*Mycobacterium tuberculosis* (*Mtb*), the causative agent of tuberculosis, infects approximately one-third of the world’s population and is responsible for about 1.4 million deaths a year [1]. The macrophage, which is the primary phagocytic cell involved in control of *Mtb* infection, imposes numerous stresses on *Mtb*, including reactive oxygen species, reactive nitrogen species, low pH and restriction of micronutrients and macronutrients. Fusion of lysosomes with phagosomes in immunologically activated macrophages exposes intra-phagosomal *Mtb* to an acidic environment and to numerous hydrolases with acidic pH optima that can degrade microbial lipids, proteins and nucleic acids. Given that *Mtb* has no known natural host other than humans [2], with whom *Mtb* appears to have co-evolved for millions of years, *Mtb* must have developed mechanisms to combat the stresses present within the human host. In non-activated macrophages, *Mtb* is capable of blocking the fusion of phagosomes with lysosomes and avoids a degradative state [3]. Activation of macrophages with interferon-gamma (IFNγ) overcomes the block in phagosome maturation and leads to acidification of the compartment to pH∼4.5 [4], [5].

The mechanisms used by *Mtb* to resist acidic stress are not well understood, as few acid-sensitive *Mtb* mutants have been identified. *Mtb* deficient in MgtC, a putative magnesium transporter, is attenuated for growth *in vitro* under mildly acidic conditions with low Mg^2+^ concentrations [6]. *Mtb* mutants lacking OmpATb, an acid-dependent protein, are sensitive to low pH conditions *in vitro* and are attenuated in macrophages [7]. Recent studies indicate that OmpATb is involved in adaptation to acidic environments by mediating secretion of ammonia, which functions to neutralize the extracellular environment [8]. The *ompATB* operon, however, is not required for virulence in mice, suggesting that *Mtb* has multiple mechanisms to resist the stress imposed by phagosomal acidification.

A screen of 10,100 *Mtb* transposon mutants identified 21 mutants that were hypersusceptible to a medium buffered to pH 4.5 [9]. Killing of *Mtb* at pH 4.5 was largely dependent on the medium used: most of the mutants displayed enhanced susceptibility in 7H9 medium containing albumin and the dispersal agent Tween 80; however, at low pH, both Tween 80 and albumin can release fatty acids, which are toxic to *Mtb* at low pH [10]. When sodium phosphate-citrate buffer was used without albumin and Tween 80 was replaced with the non-hydrolyzable detergent Tyloxapol, wild-type *Mtb* exhibited prolonged survival at pH 4.5. Of the 21 mutants, only two, *rv3671c::Tn* and *rv2136c::Tn*, remained acid-sensitive. Both strains were unable to maintain intrabacterial pH (pH_IB_) homeostasis *in vitro* and within activated macrophages [9], [11]. Moreover, both mutants were severely attenuated in mice [9], [11], raising the possibility that pH_IB_ homeostasis is essential for *Mtb*’s growth and persistence *in vivo*.

Studies with *rv2136c* were delayed by prolonged and unsuccessful efforts to complement the phenotype of the transposon mutant with a wild-type copy of *rv2136c* and/or members of the operon of which it is a part [9], [12]. Sequencing revealed the absence of mutations in previously characterized acid sensitive genes, including *mgtC*, *ompATB*, and *rv3671c* (T. Iorger and J. Sacchettini, unpublished observations), suggesting that the acid sensitive phenotype of this strain may be due to a mutation in an unidentified gene. The gene *rv3671c*, recently named *marP* for mycobacterial acid resistance protease [13], encodes a membrane-associated serine protease with the C-terminal protease domain located within the periplasm [9]. The purified extracellular domain exhibits autoproteolytic activity and is capable of cleaving β-casein and select oligopeptides [13], demonstrating that it can function as a protease; however, no substrates have been identified that are known to be involved in its biologic function [14]. While *marP* is important for pH_IB_ homeostasis in *Mtb*, additional, *marP*-independent pathways of pH_IB_ homeostasis are likely to exist.

To identify pathways involved in *Mtb’s* acid resistance, we developed a whole-cell, high-throughput screen (HTS) for compounds that interfere with pH_IB_ homeostasis in *Mtb*. *Mtb* expressing a pH-sensitive, ratiometric GFP (pHGFP) [9], [15] was suspended in an acidic buffer and used to monitor pH_IB_ in response to treatment with compounds from a natural product library. To our knowledge, this is the first documented whole-cell screen for disruptors of intrabacterial pH homeostasis, a pathway with physiologic relevance.

We identified 24 active compounds (“hits”) in the primary screen. After extensive counter-screening with liposomes, gramicidin channels, mammalian epithelial cells, erythrocytes and the lack of predicted deficiencies from a medicinal chemistry perspective (“structural alerts”), four were selected for further characterization. One of the compounds, agrimophol, further disrupted intrabacterial pH homeostasis in the *marP::Tn* and *rv2136c::Tn* mutants, suggesting that it targets different pathways for pH_IB_ homeostasis than those affected by these mutations. Such compounds may serve as tools to identify new pathways in bacterial pH control and may reveal novel targets for anti-tuberculosis chemotherapy.

## Materials and Methods

### Strains and Media

*Mtb* H37Rv (ATCC) transformed with a pH-sensitive ratiometric GFP (H37Rv-pHGFP) [15] and the similarly transformed *marP* transposon mutant (*marP::Tn-*pHGFP) were used. Both strains were cultivated in Middlebrook 7H9 at pH 6.6 with 0.2% glycerol, 0.5% bovine serum albumin, 0.2% dextrose, 0.085% NaCl and 0.05% Tween-80 (7H9) or on Middlebrook 7H11 or 7H10 plates containing 0.5% glycerol and 10% OADC (oleic acid, albumin, dextrose, catalase supplement) (Difco). For screening, strains were washed and resuspended in sodium phosphate citrate buffer pH 4.5 with 0.02% Tyloxapol (Pcit-Tyl-4.5). Sodium phosphate citrate buffer pH 7.4 with 0.02% Tyloxapol was used where indicated (Pcit-Tyl-7.4).

### Compounds

Monensin, isoniazid (INH), ethambutol (EMB), pyrazinamide (PZA), rifampin (RIF), and streptomycin (SM) were purchased from Sigma. The 1,980 natural product compounds screened were purchased from Analyticon Discovery (Potsdam, Germany), also the source of resupply. The vesicle-forming lipid 1,2-dierucoyl-*sn*-glycero-3-phosphocholine (DC_22∶1_PC) was from Avanti Polar Lipids (Birmingham, AL). Gramicidin was the natural mixture of gramicidins from *Bacillus brevis* (Sigma-Aldrich, St. Louis, MO), which is ∼80% [Val^1^]gramicidin A (gramicidin A with first amino acid as Val) (Abo-Riziq et al., 2006); the mixture is denoted as gD (for R. Dubos, who discovered the gramicidins (Dubos, 1939)).

### HTS assay

Compounds in dimethylsulfoxide (DMSO) were robotically dispensed into black-walled, clear-bottom 96-well plates containing 50 µL of Pcit-Tyl-4.5 buffer to achieve a final compound concentration of 6.25 µg/mL (∼12.5 µM) with a final DMSO concentration of 0.25%. H37Rv-pHGFP and *marP::Tn-*pHGFP were grown to mid-log phase in 7H9, washed twice, resuspended in Pcit-Tyl-4.5 to optical density (OD_580 nm_) = 0.14 and dispensed in 150 µl per well with multi-channel pipettors to a final OD_580 nm_ = 0.1. The negative control was H37Rv-pHGFP exposed to vehicle (DMSO) alone. The positive controls were *marP::Tn-*pHGFP and H37Rv-pHGFP exposed to 6.92 µg/mL (10 µM) monensin, a sodium/hydrogen ionophore. Positive and negative controls were included on each plate in triplicate. Plates were incubated at 37°C for 4, 24 and 48 hours, the fluorescence of each well was read on a tunable Molecular Devices SpectraMax M5 spectrofluorometer at excitation 395 nm, emission 510 nm and at excitation 475 nm, emission 510 nm. pH_IB_ was measured using the ratio of reading 1 (excitation 395 nm) to reading 2 (excitation 475 nm) and converting to pH_IB_ in reference to a calibration curve. The library was screened in singlet due to the limited amount of compounds; each hit was tested in duplicate and then resupplied or synthesized for further studies. HTS data were uploaded into the Collaborative Drug Discovery database for analysis. Z-factor values were calculated for each plate from the positive (*marP::Tn-*pHGFP) and negative (H37Rv-pHGFP plus DMSO) controls. For technically acceptable plates (Z-factor value ≥0.7), results for each well were plotted for pH_IB_ at each time point. Any compound which resulted in a decrease in pH_IB_ of *Mtb* to ≤pH 6.5 was considered a hit compound. This cutoff was chosen because a pH_IB_ reading of 6.5 is followed by a 1.5 log_10_ reduction in colony forming units (CFU) when the *marP::Tn* mutant is incubated in Pcit-Tyl-4.5 for six days, and because the assay is insensitive to pH values below pH 5.5.

### Screening of Conventional Antibiotics in HTS Conditions

Conventional antibiotics were screened in the above conditions using 10 2-fold dilutions of each compound, with at least 4 concentrations within the reported minimal inhibitory concentration (MIC) ranges for these drugs against *Mtb* [16]. The concentration ranges for each antibiotic tested were the following: INH (0.8 µg/mL to 1.5 ng/mL), PZA (240 µg/mL to 0.46 µg/mL), RIF (2 µg/mL to 3.9 ng/mL), EMB (30 µg/mL to 58 ng/mL), and SM (8 µg/mL to 7.8 ng/mL). A concentration-response curve for monensin was also included (28 µg/mL to 0.05 µg/mL). All antibiotics were dissolved in DMSO except for INH and SM, which were dissolved in distilled H_2_O.

### Survival Assays

Mid-log phase cultures were washed with Pcit-Tyl-4.5 or Pcit-Tyl-7.4 buffer and centrifuged at 120 g for 10 minutes. Single-cell suspensions were adjusted to an OD_580_ of 0.1 and incubated at 37°C with or without compound. After two and six days, samples were serially diluted and plated on 7H11 agar. CFUs were enumerated after two weeks. H37Rv-pHGFP treated with DMSO was used as a negative control.

### Counter-screen for Protonophoric Activity

The fluorophore fluorescein-5-(and-6-)-sulfonic acid, trisodium salt (FS) (Invitrogen) was loaded into large unilamellar vesicles (LUV) using hydration/mini-extrusion. For each batch of LUVs, 200 µL of 1,2-dierucoyl-*sn*-glycero-3-phosphocholine in chloroform (25 mg/mL) (Avanti Polar Lipids) was dried under nitrogen and further dried in a dessicator overnight to remove chloroform. The dried lipid film was rehydrated in 120 mM NaNO_3_, 10 mM FS, 10 mM Bis-Tris buffer (pH 7.4) for 4 hours at room temperature. The hydrated lipid solution was sonicated for 1 minute, subjected to 5–6 freeze thaw cycles, and extruded ∼19 times using an Avanti mini-extruder (Avanti Polar Lipids) with a 0.1 µm polycarbonate membrane filter. Extravesicular FS was removed using a PD-10 desalting column (GE Healthcare). The stock lipid solution was stored in the dark at 12.5°C for up to seven days. For experiments, the stock lipid solution was diluted 1∶50 in 140 mM NaNO_3_ plus 10 mM Bis-Tris buffer (pH 7.4). Compounds were incubated in a solution of 140 mM NaNO_3_ plus 10 mM Bis-Tris buffer (pH 4.0) for 10 minutes at 25°C. Mixing of the diluted lipid solution and the compound solution in 1∶1 ratios produced an extravesicular pH of 6.4. Each compound was tested at two concentrations (0.5 and 5 µM) with final DMSO concentrations not exceeding 0.2%. Monensin was used as a positive control (0.5 and 5 µM) and DMSO alone as a negative control. We measured FS fluorescence in an SX.20 stopped-flow spectrometer (Applied Photophysics) with a 150-W xenon lamp and a 2-sample rapid mixing unit with a machine dead time of ∼1.2 ms and an integrated water bath, with excitation at 492 nm and a 515 nm high-pass emission filter. For each sample, at least five mixing trials were performed, each with two independent vesicle preparations. Fluorescence was measured over a 5-second time course, taking 800 measurements per second. Change in fluorescence was calculated by subtracting the relative fluorescence units (RFU) at 5 s from the initial RFU at 1.25 ms. Any change in fluorescence due to DMSO alone was subtracted. Any compound that resulted in >10% decrease in fluorescence (over 5 s) was considered to disrupt the resistance of the lipid bilayer to influx of hydrogen ions and was excluded from further analysis.

### Counter-screen for Bilayer-perturbing Effects

The fluorophore 8-aminonaphthalene-1,3,6-trisulfonate (ANTS) was loaded into large unilameller vesicles (LUVs) using hydration/mini-extrusion [17], [18]. For each batch of LUVs, the lipid and gA solution was dried under nitrogen and dried further in a desiccator under vacuum overnight. The lipid film was hydrated in 100 mM NaNO_3,_ 25 mM ANTS (Na^+^ salt), 10 mM HEPES, pH 7.0 at room temperature overnight, adjusting the volume to give a 10 mM lipid with 5.2 µM gA suspension. The suspension was sonicated at low power for 1 min, subjected to five freeze-thaw cycles and extruded 21 times at room temperature using an Avanti mini-extruder with a 0.1 µm polycarbonate membrane filter. Unencapsulated ANTS was removed using a PD-10 desalting column (GE Healthcare, Piscataway, NJ), and the vesicle stock solution (∼5 mM lipid) was stored in the dark at 12.5°C for a maximum of seven days. For the fluorescence experiments, the ANTS-loaded LUV stock solution was diluted 1∶20 with extravesicular buffer solution (140 mM NaNO_3_, 10 mM HEPES at pH 7.0). From electron microscopy imaging (data not shown) the vesicle size is normally distributed with an average vesicle diameter of ∼150±50 nm.

The time course of ANTS fluorescence quenching was measured at 25°C using a SX.20 stopped-flow spectrofluorometer (Applied Photophysics, Leatherhead, UK) with a 150 W Xenon lamp and two-sample-rapid mixing unit with machine dead time ∼1.5 ms. The excitation was at 352 nm and the fluorescence was recorded above 455 nm with a high pass filter and Prodata control software from Applied Photophysics, with a sampling rate of 5000 points/s. For each sample the additives (10 µM) were incubated with the LUV suspension for 10 min and several (at least 5), 1 s mixing trials were recorded. In each buffer (fluorescence baseline) trial, the vesicle solution was mixed with extravesicular solution buffer (140 mM NaNO_3_, 10 mM HEPES at pH 7.0). In each quenching trial the vesicle solution was mixed with quenching buffer (50 mM TlNO_3_, 94 mM NaNO_3_, 10 mM HEPES at pH 7.0).

To quantify the drug-dependent changes in the time course of fluorescence quenching, the kinetics of ANTS quenching by Tl^+^ must be considered. For a single vesicle with a fixed number of conducting channels, the Tl^+^ influx can be approximated as a first-order process. The size distribution of the vesicles and the distribution of channel lifetimes, however, lead to different-sized vesicles filling with Tl^+^ at varying rates–and initially active pores may deactivate and new channels form over the time course of the experiment. The resulting fluorescence quenching time course therefore becomes a complex, multi-exponential function that varies with the vesicle size distribution and channel formation rate and lifetime. The time course can be fit empirically by a stretched exponential [19]:
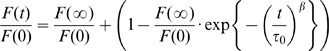
(1)where β is a parameter that depends on the LUV dispersity (0< β ≤1) and τ_0_ a rate parameter with units of time.

For the analysis, the fluorescence intensities were normalized to the initial (first 2–10 ms) average fluorescence in the absence of quencher for the specific sample. For each experiment, the initial 2–100 ms of each quenching repeat were fitted by Eq. 1 using a nonlinear least squares fit implemented in MATLAB (The MathWorks, Natick, MA), which provides estimates for *F*(0), F(∞), β and τ_0_. The initial influx rate was then estimated as the fluorescence quench rate *k*(*t*):
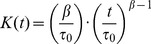
(2)evaluated at 2 ms [17], which provides a robust measure of changes in gA activity that corresponds to the results of electrophysiological measurements of changes in gA activity.

### Hemolysis Assay

Blood was collected from normal human donors in heparinized tubes under an IRB approved protocol. Isolated human red blood cells (RBC) were collected from the bottom of Ficoll-Paque gradients [20], washed three times with 2 volumes of phosphate-buffered saline (PBS [no Ca^2+^, Mg^2+^]) by centrifugation at 500 g for 5 min and resuspended in PBS at 2 times the starting volume of blood. RBC (100 µL) were dispensed in Costar 96-well round-bottomed plates. To each well, 100 µL of PBS or 100 µL of distilled water (positive control) was added to achieve a total well volume of 200 µL. RBC were treated with 12.5 µM of compound in DMSO (final concentration of DMSO, 0.25%) or 0.25% DMSO alone as a negative control. Samples were incubated at 37°C for 1 hour. Plates were centrifuged at 500 g for 5 minutes and 100 µL of the supernatant was removed and diluted five-fold. 200 µL of the diluted supernatant was transferred to a Costar 96-well black walled clear-bottomed plate and absorbance was read in a spectrophotometer at 560 nm to evaluate hemoglobin release. Any compound that resulted in ≥5% lysis of RBC was discarded.

### Mammalian Cell Toxicity

Vero green monkey kidney cells (ATCC) were cultured in Dulbecco’s modified Eagle’s medium with 10% FBS, 100 units/mL penicillin, 100 mg/L streptomycin, 0.5 mg/L gentamicin, 2 mM L-glutamine, 1 mM sodium pyruvate and 1 mM HEPES. Confluent cells were trypsinized, counted in a hemocytometer, and seeded in 200 µL in 96 well plates at 1×10^4^ cells/well and incubated at 37°C for two days. The medium was removed, the cells washed once with PBS and medium containing 2% FBS and compound was added (final DMSO, 0.25%). After two days at 37°C viability was assessed microscopically and by a tetrazolium (MTS) reduction assay (Promega).

### Membrane Potential Analysis

Alterations in membrane potential were determined using the membrane potential-sensitive cyanine dye DiOC_2_ (Invitrogen) as described [21]. In brief, *Mtb* cultures were grown to mid-log phase (OD_580 nm_∼0.6) and resuspended and concentrated to an OD_580 nm_∼1.0 in Pcit-Tyl–4.5 or Pcit-Tyl–7.4. Compound was added to cultures to achieve final concentrations of 6.25, 12.5 or 25 µM. Cultures were immediately exposed to 15 µM of 3,3′-diethyloxacarbocyanine iodide [DiOC_2_] (Invitrogen) for 20 minutes at room temperature. Bacteria were washed to remove extracellular dye and resuspended in fresh buffer. As a positive control for membrane depolarization, 5 µM of the protonophore carbonyl-cyanide 3-chlorophenylhydrazone (CCCP) (Invitrogen) was included. DMSO and RIF (0.4 µg/mL) were used as negative controls. The assay was performed using clear-bottom, black 96-well plates (Costar) in a Molecular Devices SpectraMax M5 spectrophotometer. We measured green fluorescence (488 nm/530 nm) and shifts to red fluorescence (488 nm/610 nm) as a result of aggregation of dye molecules due to the presence of a large membrane potential. Membrane potential was measured as a ratio of red fluorescence, which is cell size-dependent and membrane potential-dependent, to green fluorescence, which is cell size-dependent and membrane potential-independent. Each condition was measured in triplicate and each experiment was performed twice.

### Synthesis of Compound 1048

This is described in File S1.

### Extraction and Purification of Natural Agrimophol

All materials used were of commercial grade without purification unless otherwise specified. The hairyvein agrinonia rhizome was purchased from An Guo medicinal materials market in Hebei province of China. The nuclear magnetic resonance (NMR) experiments were carried out on a Varian Mercury 300 MHz or 600 MHz spectrometer in CDCl_3_. Chemical shifts were reported in ppm (*δ*) relative to the solvent, and coupling constants (*J*) were reported in Hz. Melting points were determined without correction with a Yanaco micromelting point apparatus. HPLC was conducted on an Agilent Technology 1200 series system equipped with an Agilent pump, an Agilent detector and an Agilent liquid handler. The column used was an Eclipse XDB-C18 column (5.0 µm, 4.6 mm×150 mm) from Agilent with a flow rate of 1.0 mL/min for analysis. The UV detection was carried out at a UV wavelength of 254 nm. High resolution LC-MS (HRMS) was carried out by Agilent LC/MSD TOF using a column of Agilent ZORBAX SB-C18 (rapid resolution, 3.5 µm, 2.1 mm×30 mm) at a flow of 0.40 mL/min. The solvent is methanol/water = 75∶25 (v/v) containing 5 mmol/L ammonium formate. The ion source is electrospray ionization (ESI).

The hairyvein agrinonia rhizome (1 kg) was soaked in 10 L of petroleum (60–90°C) for 48 hours at room temperature. The solvent was concentrated in vacuo to yield a crude extract, which was then dissolved in chloroform (30 ml). After removing the insoluble substance, the filtrate was concentrated in vacuo to yield a paste, which was then dissolved in petroleum in a clear beaker flask and standing at room temperature. After a precipitate formed, the mixture was filtered and the solid was subjected to recrystallization. The above operations were repeated several times until light yellow crystals were obtained. Finally we obtained 58 mg of pure agrimophol as light yellow powder in 0.0058% yield; mp 138.5–140°C. ^1^H NMR (300 MHz, CDCl_3_) δ 19.25 (s, 1H), 15.55 (s, 1H), 11.07 (s, 1H), 9.63 (s, 1H), 3.91–3.82 (m, 1H), 3.78 (s, 3H), 3.22–3.02 (m, 2H), 3.14–2.93 (m, 2H), 2.20 (s, 3H), 1.91 (s, 3H), 1.82–1.66 (m, 3H), 1.48–1.34 (m, 1H), 1.18 (d, *J* = 6.9 Hz, 3H), 1.05 (s, 3H), 1.01 (t, = 7.5 Hz, 3H), 0.88 (t, = 7.2 Hz, 3H). ^13^C NMR (125 MHz, CDCl_3_): δ 209.7, 207.1, 200.9, 190.3, 174.2, 163.0, 160.7, 160.4, 112.3, 107.5, 106.2, 104.5, 104.2, 61.6, 52.6, 44.2, 43.0, 27.8, 26.5, 18.1, 16.8, 13.9, 11.8, 9.1, 7.0. HRMS calcd for C_26_H_35_O_8_ [M+H]^+^: 475.2326; found: 475.2337.

## Results

### High-throughput Screen

In order to identify inhibitors of pH_IB_ homeostasis, we used an *Mtb* strain expressing a pH-sensitive, ratiometric GFP (pHGFP) [9], [15] to monitor changes in pH_IB_ in response to treatment with compounds. To mimic the acidic environment of the phagolysosomal compartment, compounds were screened against *Mtb* at pH 4.5. We used phosphate citrate buffer supplemented with Tyloxapol at pH 4.5 (Pcit-Tyl–4.5) instead of traditional 7H9 complete medium in order to avoid the toxic affects of fatty acids released from Tween and albumin at low pH [9]. The assay is insensitive to pH_IB_ changes below 5.5, and a pH_IB_ reading of 6.5 is followed by a 1.5 log_10_ reduction in colony forming units (CFU) when the *marP::Tn* mutant is incubated in Pcit-Tyl–4.5 for six days [9]. Therefore, any compound that resulted in a decrease in *Mtb* pH_IB_ to ≤ 6.5 was considered to be a hit compound. As a genetic positive control, we used the *marP::Tn* mutant. As a chemical positive control, we used the protonophore monensin.

**Figure 1:**
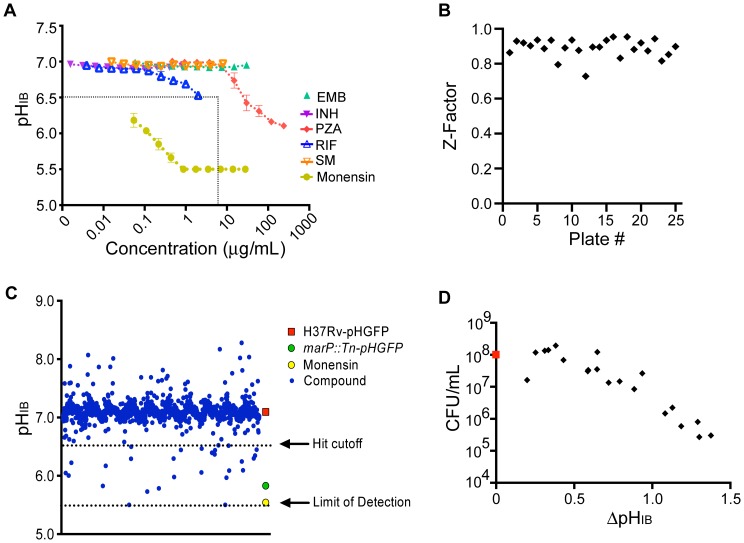
Effects of antibiotics, monensin, and other natural products on pH_IB_ of *Mtb*. (A) H37Rv-pHGFP was treated with vehicle control DMSO, antibiotic, or monensin in phosphate citrate buffer pH 4.5 and pH_IB_ measurements were taken after two days of exposure. pH_IB_ of *marP::Tn-*pHGFP dropped to pH 5.5 under the assay conditions. Dotted black lines indicate hit cutoff pH and HTS screening concentration. Means ± standard deviations of triplicate experimental samples are shown from one experiment, representative of three. (B) Z-factor values were calculated from DMSO treated H37Rv-pHGFP and *marP::Tn-*pHGFP controls located on each plate screened. (C) pH_IB_ measurements for H37Rv-pHGFP exposed to 1,980 natural products and controls after two days at pH 4.5. (D) Correlation between change in pH_IB_ and survival of *Mtb-*pHGFP after a two day exposure to 20 hit compounds at pH 4.5. Results represent two independent experiments, each performed in duplicate. Untreated, DMSO control in red.

To evaluate whether the screening conditions would select for compounds with similar mechanisms of actions as conventional anti-tuberculosis antibiotics, we tested isoniazid (INH), ethambutol (EMB), pyrazinamide (PZA), rifampin (RIF), and streptomycin (SM) in our assay conditions. Only PZA decreased pH_IB_ to below 6.5 within 48 hours (Figure 1A), perhaps because pyrazinoic acid (POA) could shuttle protons from the extracellular space into the intrabacterial space. The highest concentrations tested of RIF also decreased pH_IB_, but not below 6.5. None of the remaining compounds had any effect on pH_IB_ at the concentrations tested, indicating that this assay may select for compounds with novel modes of action or with mechanisms similar to PZA, which is perhaps the most efficient sterilizing drug available for the treatment of tuberculosis. The chemical control, monensin, affected pH_IB_ at concentrations as low as 0.05 µg/mL.

Next, we screened 1,980 natural products against *Mtb* H37Rv at 6.25 µg/mL (∼12.5 µM). A Z-factor was calculated for each screening plate using the DMSO-treated H37Rv negative control and the *marP::Tn* positive control. The Z-factor is a statistical parameter that provides a measure of the assay’s signal to noise performance by evaluating the separation in means and standard deviations of the positive and negative controls; the maximal value is 1.0. A HTS assay is considered to have good discovery potential if it has a Z-factor >0.5. In our screen, Z-factor values had a mean ± standard deviation (SD) of 0.89±0.06 and were > 0.7 for all plates (Figure 1B). Twenty-four compounds (1.2%) resulted in a decrease in pH_IB_ to ≤6.5 (Figure 1C). These 24 “hits” were re-screened at 6.25 µg/mL in duplicate experiments; 22 compounds were active in both assays, giving a 92% confirmation rate.

Of the 22 confirmed active compounds, 20 were re-supplied; two were not available. The 20 re-supplied compounds were re-tested at 6.25 µg/mL to confirm their effects on pH_IB_ and evaluated for their effects on survival of *Mtb* at pH 4.5. Disruption of pH_IB_ correlated directly with a mycobactericidal effect (Figure 1D). Five compounds were less potent than originally observed and were re-tested at 25 µM. Four of these five compounds fell within our cut off range at 25 µM and proceeded through to secondary screens, while the remaining compound was eliminated. The structures of the 19 confirmed hits are shown in figure S1.

### Secondary Screens

**Figure 2:**
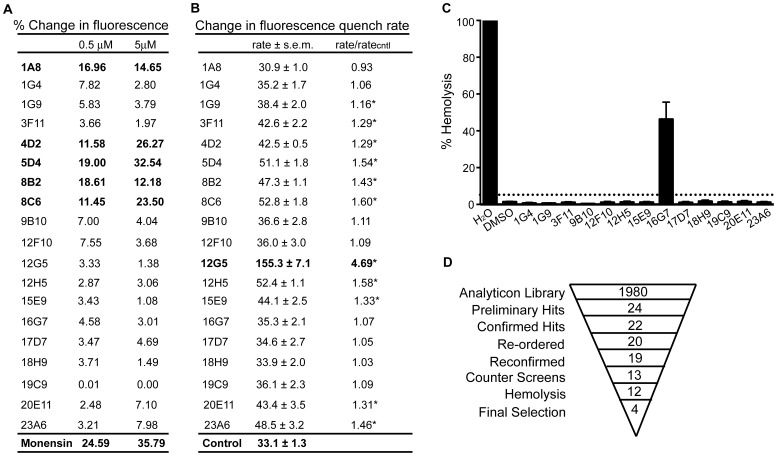
Secondary screens and summary of HTS results. (A) Liposome assay: large unilamellar vesicles were loaded with fluorescein-5-(and-6-)-sulfonic acid, trisodium salt at pH 7.4 and exposed to pH 6.4 with or without compound. Fluorescence was measured over a 5 second time course, and numbers are adjusted for DMSO control. (B) Gramicidin channel assay: ANTS-loaded large unilamellar vesicles were mixed with quenching buffer and compound (10 µM) and the rate of fluorescence quenching was measured. *n = *7–8 for each compound and 33 for control (DMSO only). *denotes that the *p* value relative to control is less than 0.001. (C) Human red blood cells were exposed to compound for 1 hour and absorbance was read in a spectrophotometer at 560 nm to evaluate heme release. Dotted black line represents 5% lysis cutoff. Results show means ± SEM of two independent experiments, each performed in duplicate. (D) Summary of screening results.

Compounds can disrupt pH_IB_ homeostasis by acting as protonophores or by perturbing the bacterial membrane. Neither property would make such compounds useful tools to identify specific gene products involved in regulating pH_IB_. To eliminate such compounds, we developed a liposome counter-screen for protonophoric activity (Figure S2). We loaded liposomes with a pH-sensitive dye, fluorescein-5-(and 6-) sulfonic acid (FA) at neutral pH and then subjected the liposomes to a slightly acidic pH (6.4) with or without the test compound. Compounds that disrupt lipid bilayers or that carry protons across them will produce a rapid decrease in fluorescence due to a drop in intraliposomal pH. Of the 19 compounds tested, 5 (1A8, 4D2, 5D4, 8B2, 8C6) resulted in a >10% decrease in fluorescence over a 5 s exposure to pH 6.4 (Figure 2A). One of these, lasalocid (5D4), is a known ionophore that is chemically related to the positive chemical control, monensin.

Because many of the hits are rather hydrophobic/amphiphilic, they have the propensity to adsorb at the membrane or solution interface and thereby alter lipid bilayer properties [22], and thus be promiscuous modifiers of membrane protein function. As a complement to the liposome assay, we therefore employed a gramicidin channel assay to detect compounds with membrane-perturbing properties [17], [18]. The assay uses the ion-conducting gramicidin channels that form by trans-membrane dimerization of two monomers from opposing leaflets of the bilayer. The gramicidin monomer↔dimer equilibrium is sensitive to the membrane environment, making the gramicidins suitable to assay for membrane-perturbing effects. The bilayer-spanning gramicidin channels allow for the entry of monovalent heavy-ion quenchers, and the consequent quenching of fluorophore-loaded large unilamellar vesicles (LUV) (Figure S3). The rate of fluorescence quenching is proportional to the number of conducting gramicidin channels, which will vary based on the membrane-perturbing effects of the added compounds. We measured the time course of fluorescence quenching in the presence of compound using the 8-aminonaphthalene-1,3,6-trisulfonate (ANTS)/Tl+ fluorescence indicator/quencher pair. While more than 50% of the compounds produced a statistically significant increase in the fluorescence quench rate, one compound, 12G5, had a pronounced effect (Figure 2B) and was eliminated. Together, the liposome and gramicidin channel assay counter-screens eliminated six compounds (1A8, 4D2, 5D4, 8B2, 8C6 and 12G5) from further studies.

Next, we used a hemolysis assay to further evaluate membrane-disrupting potential or other cytotoxic properties against mammalian cells. One compound (16G7; Figure 2C) caused hemolysis and was excluded.

**Figure 3:**
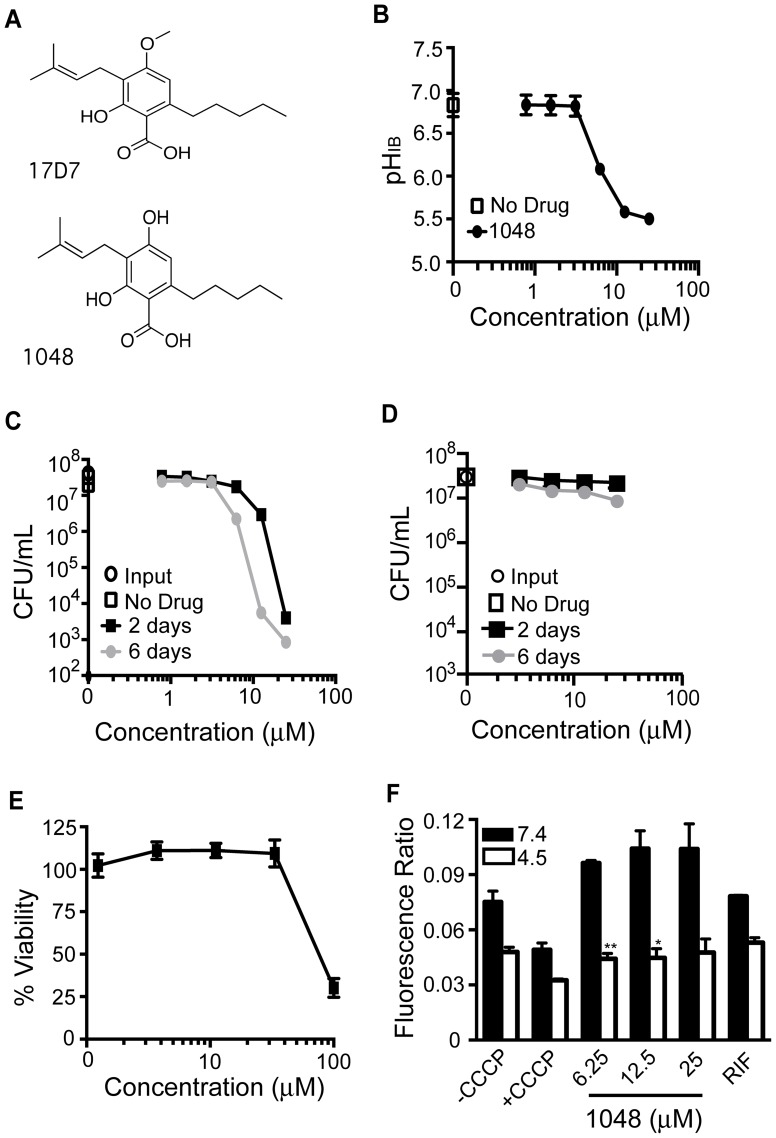
Effect of 17D7 and 1048 on pH_IB_, survival, membrane potential of *Mtb* and on toxicity to Vero green monkey kidney cells. (A) Structure of 17D7 and 1048. (B) pH_IB_ was measured following exposure of H37Rv-pHGFP to increasing concentrations of 1048 at pH 4.5 for two days at 37°C. (C and D) Survival of H37Rv-pHGFP following exposure to increasing concentrations of 1048 at pH 4.5 (C) and pH 7.4 (D) for two and six days. Means ± SEM of triplicate samples from two experiments are shown. (E) Vero cells were exposed to varying concentrations of 1048 for two days at 37°C and viability assessed microscopically and by a tetrazolium (MTS) reduction assay. Results represent means and standard deviations for two independent experiments, each performed in triplicate. (F) H37Rv treated with DMSO, CCCP, RIF (0.4 µg/mL), or 1048 at pH 7.4 or 4.5 was exposed to the membrane potential sensitive dye, DiOC_2_. Results represent means and standard deviations for two independent experiments, each performed in triplicate. The p value was determined using an unpaired t test: **p = 0.0045; *p = 0.029.

The structures of the remaining 12 compounds were then inspected for potentially reactive groups, likely modifications in the human body that might generate reactive groups, and other features that might make the compounds non-selective as a starting point to construct chemical probes. The coumarin scaffold in compound 1G4 is associated with diverse pharmacologic actions [23], which might complicate its use for target identification. Compound 3F11, an anthraquinone, was eliminated because of the potential of this chemophore to generate reactive oxygen species and to intercalate in DNA [24], [25]. Compounds with long-chain aliphatic acids (9B10, 15E9, 18H9, 19C9) could potentially disrupt the *Mtb* membrane, allowing for entry of protons into the intracellular space, even though they had no such effect in the counter-screens. Compounds 12F10 and 12H5 contain reactive aldehyde moieties that are likely to allow them to bind to diverse proteins as well as DNA; additionally, 12H5 caused more than a 1.5-fold increase in fluorescence quench rate (Figure 2A). Supplies of compound 17D7 were insufficient to support more detailed studies. Efforts to synthesize 17D7 in-house were unsuccessful at producing the final product, but generated the closely related compound 1048 (Figure 3A), in which a phenolic hydroxyl replaces the methoxy in 17D7. Compound 1048 was 2- to 4-fold more potent than 17D7 in the pH_IB_ assay (Figure S4) and was used in subsequent studies. A summary of the selection process is depicted in figure 2D.

### Characterization of Selected Actives

Among the top four hit compounds (1048, 2, 4-dihydroxy-3-(3-methylbut-2-enyl)-6-pentylbenzoic acid; 20E11, 2-hydroxy-3-(2-hydroxy-3-methylbut-3-enyl)-4-methoxy-6-(2-phenylethyl)benzoic acid; 1G9, (2Z)-2-(3-hydroxy-5-oxo-4-pentylfuran-2-ylidene)acetic acid; and 23A6, 6-(3-butyryl-2, 6-dihydroxy-4-methoxy-5-methylbenzyl)-3, 5-dihydroxy-4, 6-dimethyl-2-(2-methylbutanoyl)cyclohexa-2, 4-dien-1-one), three contained a carboxylate functionality. Although the final hit compounds were enriched in carboxylates, there was no correlation between the number of carboxylates or hydroxyls and the pH_IB_ of *Mtb* incubated with the compounds tested from this library (Figure S5). The four hit compounds were further characterized for their concentration-dependent effects on *Mtb* pH_IB_, survival of *Mtb* at pH 4.5 and pH 7.4, viability of Vero cells (green monkey kidney fibroblasts), and *Mtb’s* membrane potential (ΔΨ).

On exposure to pH 4.5 and compound 1048 at 6.25 µM, the pH_IB_ of *Mtb* decreased to 6.0 after 48 hr; pH_IB_ was further decreased to the limit of detection (5.5) at 12.5 and 25 µM (Figure 3B). Mycobactericidal effects of compound 1048 were concentration- and time-dependent (Figure 3C). After six days of exposure at pH 4.5, compound 1048 (25 µM) resulted in ∼4 log_10_ reduction in CFU (Figure 3C), but there was little impact on *Mtb’s* survival at pH 7.4 (Figure 3D). While compound 1048 was nontoxic to Vero cells at concentrations up to 25 µM, a 4-fold higher concentration did reveal mammalian cell cytotoxicity (Figure 3E). The mechanism of disruption of pH_IB_ did not appear to be related to a decrease in ΔΨ of *Mtb* (Figure 3F).

**Figure 4:**
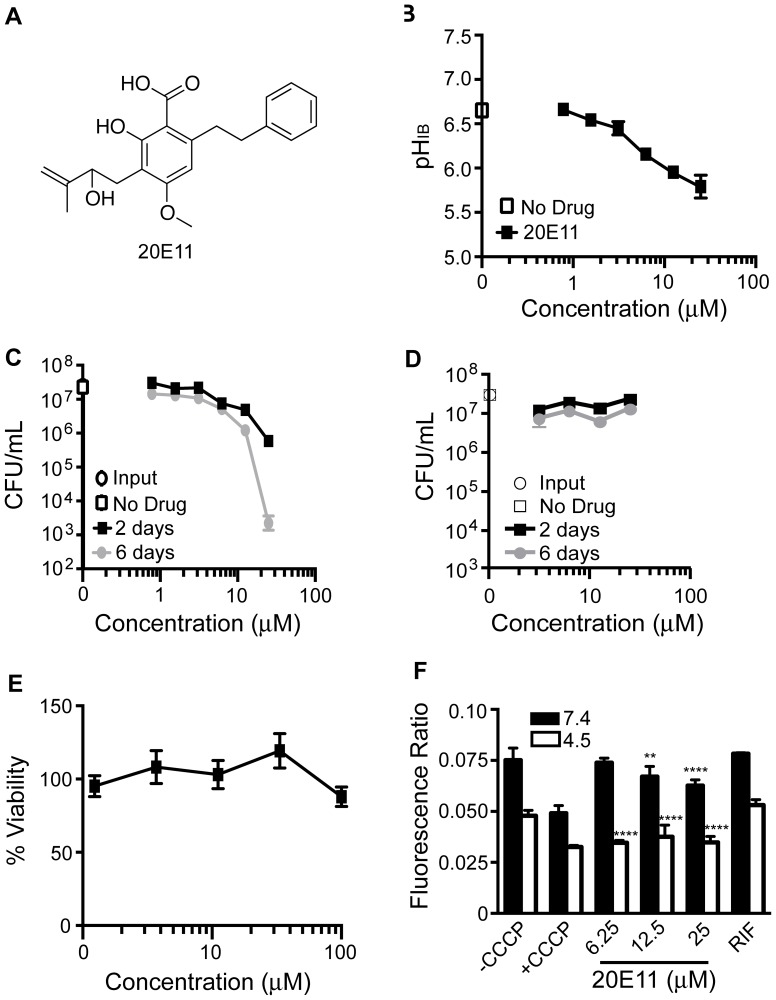
Effect of 20E11 on pH_IB_, survival, membrane potential of *Mtb* and on toxicity Vero green monkey kidney cells. (A) Structure of 20E11. (B) pH_IB_ was measured following exposure of H37Rv-pHGFP to increasing concentrations of 1G9 for two days at 37°C. (C and D) Survival of H37Rv-pHGFP following exposure to increasing concentrations of 1G9 at pH 4.5 (C) and pH 7.4 (D) for two and six days. Means ± SEM of triplicate samples from two experiments are shown. (E) Vero cells were exposed to varying concentrations of 20E11 for two days at 37°C and viability assessed microscopically and by a tetrazolium (MTS) reduction assay. Results represent two independent experiments, each performed in triplicate. Means ± standard deviations are shown. (F) H37Rv treated with DMSO, CCCP, RIF (0.4 µg/mL), or 20E11 at pH 7.4 or 4.5 was exposed to the membrane potential sensitive dye, DiOC_2_. Results represent means and standard deviations for two independent experiments, each performed in triplicate. The p value was determined using an unpaired t test: ****p<0.0001; **p = 0.0041.

Compound 20E11 shares a 4-methoxybenzoic acid core structure with 17D7 and 1048 (Figure 4A). Like 17D7, 20E11 exhibited limited potency with a decrease in pH_IB_ to only 6.0 at the highest concentration tested (Figure 4B). Nonetheless, 20E11 severely impacted *Mtb’s* ability to survive at pH 4.5, reducing CFUs by 4 log_10_ after six days at 25 µM (Figure 4C). Selectivity of this effect was suggested by the lack of mycobactericidal activity at neutrality (Figure 4D). Toxicity towards mammalian cells was not observed up to 100 µM (Figure 4E). Compound 20E11 decreased *Mtb’s* ΔΨ at pH 7.4 in a concentration-dependent manner and decreased ΔΨ to the same extent as the positive control compound CCCP at pH 4.5 (Figure 4F).

**Figure 5:**
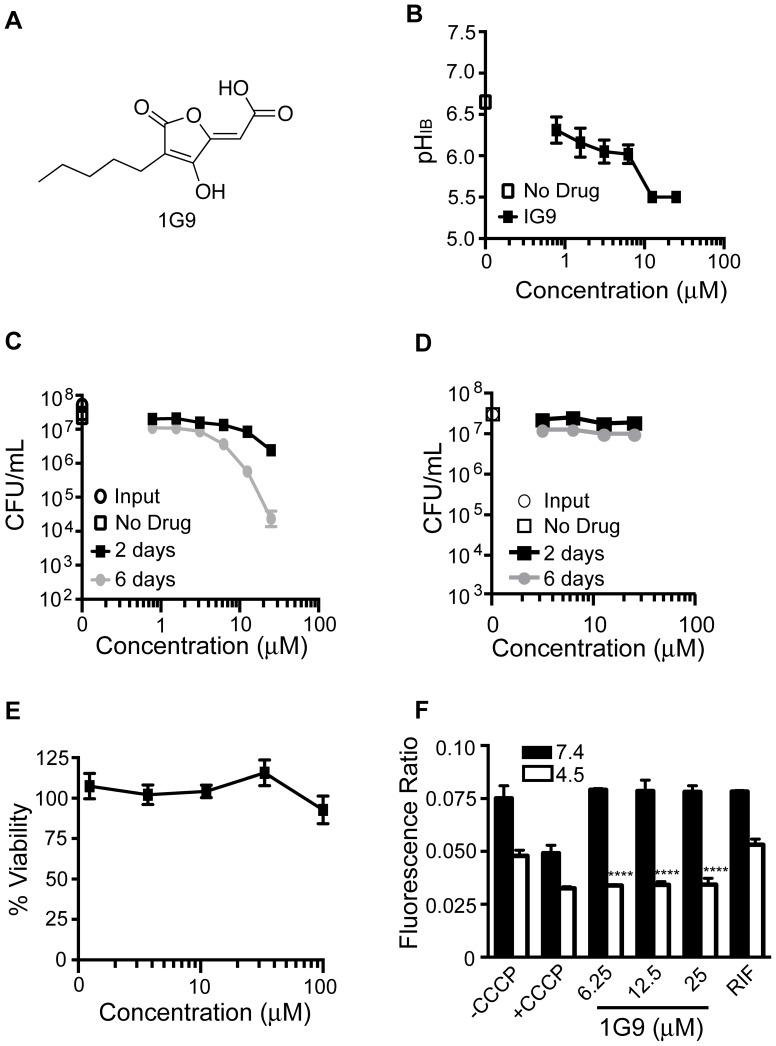
Effect of 1G9 on pH_IB_, survival, and membrane potential of *Mtb* and on Vero green monkey kidney cells. (A) Structure of 1G9. (B) pH_IB_ was measured following exposure of H37Rv-pHGFP to increasing concentrations of 1G9 for two days at 37°C. (C and D) Survival of H37Rv-pHGFP following exposure to increasing concentrations of 1G9 at pH 4.5 (C) and pH 7.4 (D) for two and six days. Means ± SEM of triplicate samples from two experiments are shown. (E) Vero cells were exposed to varying concentrations of 1G9 for two days at 37°C and viability assessed microscopically and by a tetrazolium (MTS) reduction assay. Results represent two independent experiments, each performed in triplicate. Mean ± standard deviations are shown. (F) H37Rv treated with DMSO, CCCP, RIF (0.4 µg/mL), or 1G9 at pH 7.4 or 4.5 was exposed to the membrane potential sensitive dye, DiOC_2_. Results represent means ± standard deviations for two independent experiments, each performed in triplicate. The p value was determined using an unpaired t test: ****p<0.0001.

Compound 1G9 has a 4-hydroxyfuranone core structure (Figure 5A) resembling vulpinic acid. Vulpinic acid is a secondary metabolite produced by lichens that inhibits the growth of *Mtb* with a minimum inhibitory concentration (MIC) of 64 µg/mL [26]. Compound 1G9 decreased pH_IB_ to just below the pH 6.5 cutoff at 0.78 µM (Figure 5B). As observed with compound 1048, the effects on survival at pH 4.5 were time-dependent. By six days post exposure, CFU were reduced ∼1.5 log_10_ and 3 log_10_ at 12.5 and 25 µM, respectively (Figure 5C). Mycobactericidal activity was pH-dependent, as none was observed at neutrality (Figure 5D). No toxicity toward Vero cells was evident up to 100 µM, the highest concentration tested (Figure 5E). At neutral pH, 1G9 had no effect on ΔΨ; however, at pH 4.5, 1G9 decreased ΔΨ as extensively as did CCCP (Figure 5F).

**Figure 6:**
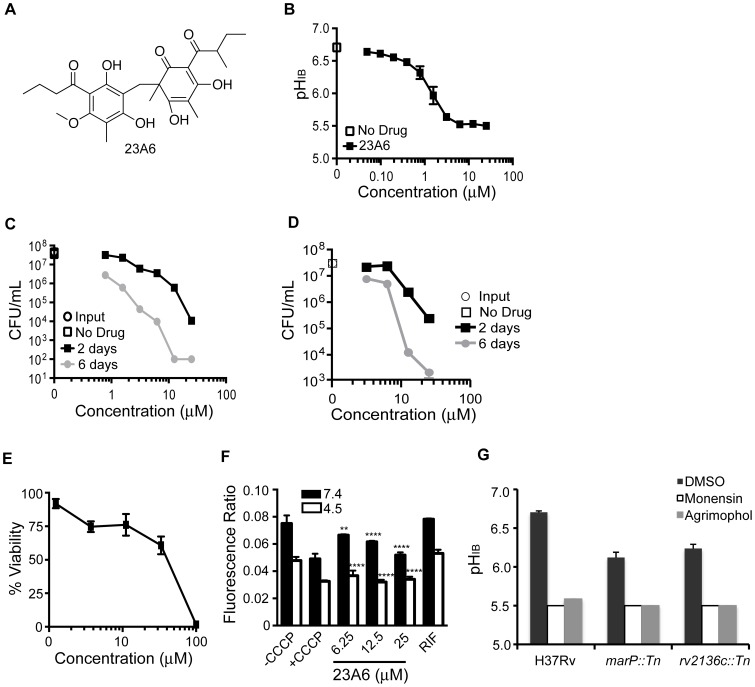
Effect of 23A6 (agrimophol) on pH_IB_, survival, and membrane potential of *Mtb* and on Vero green monkey kidney cells. (A) Structure of 23A6. (B) pH_IB_ was measured following exposure of H37Rv-pHGFP to increasing concentrations of 23A6 for two days at 37°C. (C and D) Survival of H37Rv-pHGFP following exposure to increasing concentrations of 23A6 at pH 4.5 (C) and pH 7.4 (D) for two and six days. Means ± SEM of triplicate samples from two experiments are shown. (E) Vero cells were exposed to varying concentrations of 23A6 for two days at 37°C and viability assessed microscopically and by a tetrazolium (MTS) reduction assay. Results represent two independent experiments, each performed in triplicate. Mean ± standard deviations are shown. (F) H37Rv treated with DMSO, CCCP, RIF (0.4 µg/mL), or 23A6 at pH 7.4 or 4.5 was exposed to the membrane potential sensitive dye, DiOC_2_. Results represent means ± standard deviations for two independent experiments, each performed in triplicate. (G) pH_IB_ was measured following exposure of H37Rv-pHGFP, *marP::Tn*-pHGFP, and *rv2136c::Tn*-pHGFP to purified agrimophol for two days at 37°C. Strains were treated with vehicle control DMSO, monensin (10 µM), or agrimophol at 12.5 µM in phosphate citrate buffer pH 4.5. The average and standard deviation of triplicate samples are shown. The p value was determined using an unpaired t test: ****p<0.0001; **p = 0.0014.

Compound 23A6, a phloroglucinol called agrimophol (Figure 6A), is a natural product with a history of use in traditional Chinese medicine for the treatment of helminthic and protozoal infections [27], [28]. Among the active compounds that passed our counter-screens, it is the most potent and the only one with a history of use in humans. 23A6 decreased *Mtb’s* pH_IB_ below 6.5 at 0.39 µM and below the limit of sensitivity of the assay (pH 5.5) at 3 µM (Figure 6B). Potent effects on *Mtb’s* survival at pH 4.5 were observed at low micromolar concentrations of 23A6∶0.78 µM resulted in l log_10_ and 10 µM resulted in >5 log_10_ reduction in CFU after six days (Figure 6C). 23A6 also had pronounced mycobactericidal activity at pH 7.4 (Figure 6D), although it was not as potent as at pH 4.5, suggesting that 23A6 may have a target that is critical for *Mtb*’s survival under both conditions, or different targets, each essential under one of the conditions. The concentration of 23A6 that was lethal for 50% of Vero cells (LD_50_) was ∼50 µM (Figure 6E). The compound decreased *Mtb’s* ΔΨ at pH 7.4 in a concentration-dependent manner and decreased ΔΨ to the same extent as CCCP at pH 4.5 (Figure 6F).

To determine whether 23A6, one of the most potent hits, targets the same pathways previously identified in a genetic screen [9], we determined the pH_IB_ of the *marP::Tn* and *rv2136c::Tn* mutants following exposure to 23A6. Supplies of 23A6 from the original provider were insufficient to complete this study, so we extracted natural agrimophol from the roots of *Agrimonia pilosa*, a perennial of the family Rosaceae. The agrimophol purified in-house decreased the pH_IB_ of WT H37Rv with potency comparable to that of the material from the original supplier (Figure 6G) and was used in this assay. Agrimophol decreased pHIB in both *marP::Tn* and *rv2136c::Tn* to the limit of detection (pH 5.5) after two days of incubation. This was a greater degree of intrabacterial acidification than seen with the mutants alone (Figure 6G), suggesting that agrimophol targets pathways of pHIB homeostasis other than those dependent on *marP* or the gene whose mutation is responsible for the phenotype of the *rv2136c::Tn* mutant.

## Discussion

This work describes a novel whole-cell assay suitable for a HTS format that allows identification of small molecules that perturb pH_IB_ homeostasis. Compared to other whole-cell screening assays for *Mtb,* which have incubation periods of several days or weeks, the pH_IB_ homeostasis assay is rapid, with results in as little as 4 hours, although we conducted our initial screen at two days. We have adapted this assay to a 384-well format and helped distribute it to another screening center in order to identify additional tool compounds and, potentially, precursors of lead compounds for the treatment of tuberculosis. Secondary screens eliminated compounds with protonophoric and membrane-perturbing properties.

Although we developed the assay to identify inhibitors of pH_IB_ homeostasis, the assay may also identify compounds with other activities against *Mtb.* For example, agrimophol disrupted *Mtb*’s pH homeostasis and killed *Mtb* in acidic conditions, but it also killed *Mtb* near neutrality in replicating conditions. Moreover, the assay may identify compounds that kill *Mtb* whose replication is halted not only by physiologic levels of acid but by other host-imposed stresses as well. Non-replicating subpopulations of *Mtb* are phenotypically relatively resistant to most standard chemotherapeutics used to treat tuberculosis.

To our knowledge, this is the first report of an assay for compounds that disrupt intrabacterial pH homeostasis. This may also be the first report of PZA’s effects on pH_IB_ in *Mtb*. PZA is a clinically important but paradoxical and unconventional drug. Despite its remarkable sterilizing activity *in vivo*, it is inactive against *Mtb* under standard culture conditions (in which *Mtb* is replicating) but weakly active against *Mtb* exposed to an acidic pH, conditions under which *Mtb* replicates little. Fatty acid synthase-I has been proposed as a target for PZA, but while 5-Cl-pyrazinamide targets this protein, PZA does not [29]. Recent studies point to RpsA and trans-translation as a target of pyrazinoic acid (POA) [30]. It has also been proposed that POA does not have a specific cellular target but simply functions to shuttle protons from the extracellular space into the intrabacterial space, resulting in decreased pH_IB_, collapse of membrane potential, and bacterial death [31]. Our results provide direct evidence that PZA lowers *Mtb’s* pH_IB_ in an acidic environment. This assay may select for compounds with similar sterilizing abilities as PZA, an important goal, as resistance to PZA is increasing.

We chose to screen a natural product library because of natural products’ structural diversity and greater propensity for anti-infective activity than seen with compounds produced by conventional combinatorial chemistry. A particular challenge in the chemical biology of *Mtb* is its thick cell wall comprised largely of mycolic acids and their esters. Many of the hits from this screen have a high degree of lipophilicity. Positive correlations have been observed between lipophilicity of fluoroquinolones and their efficacy against *M. leprae* [32]. However, lipophilic compounds can also have toxic effects by altering cell membrane organization and function [22], [33]. Loss of membrane integrity, for example, can dissipate trans-membrane gradients of protons and other ions. Although a given cell type in vitro may survive membrane perturbations, such disturbances often take a toll on the host. For this reason, we included several counter-screens, including the liposome assay and the hemolysis assay. Although the liposome assay is very sensitive, it does not recapitulate the properties of all types of cell membranes; for this reason, the hemolysis assay was used to reveal membrane perturbants that the liposome-based assay missed. Finally, the Vero cell toxicity assay revealed additional toxic compounds, highlighting the importance of the counter-screens in the triage. Several of the active compounds contained one or more hydroxyl or carboxyl groups, which could function to donate protons after entry into *Mtb,* resulting in decreased pH_IB_. However, we did not observe a difference between the number of hydroxyl or carboxyl groups among compounds that were active and those that were not (Figure S5), suggesting that such groups were not contributory or were not sufficient for disruption of intrabacterial pH homeostasis. Moreover, compounds with protonophoric properties should have been eliminated during the liposome counter screen, unless the difference in extraliposomal pH (6.4) and extrabacterial pH (4.5) impacted these compounds’ behavior.

We observed temporal discrepancies between decreases in pH_IB_ and effects on mycobacterial survival. It appears that *Mtb* can withstand moderate decreases in pH_IB_ for at least two days. When *Mtb* was incubated in phosphate-citrate buffer with no other carbon source and no nitrogen source at an ambient pH of 4.5, its pH_IB_ ranged from 7.0 to as low as 6.6 without any detectable impact on survival for up to six days. However, when pH_IB_ was brought lower than pH 6.5 by the compounds studied here, viability subsequently fell, often precipitously, although with a variable delay. A compound like 20E11 whose effect on viability seemed to far exceed its impact on pH_IB_ may target an important pathway in addition to one involved in pH_IB_ homeostasis. This may also be true for agrimophol, which killed *Mtb* at neutrality. Compounds 1048, 20E11, and 1G9 had no effect on survival of *Mtb* at neutral pH, which could suggest that their biological target(s) are only required/active during exposure to acid.

The inhibitors identified using the pH_IB_ homeostasis assay may advance our understanding of acid resistance mechanisms in *Mtb* and may also reveal novel targets for anti-tuberculosis chemotherapy.

### Conclusions

To our knowledge, this is the first description of a whole-cell assay for compounds that disrupt intrabacterial pH homeostasis, and of associated counter-screens to improve the likelihood that active compounds will be useful as tools to identify the molecular pathways involved. *Mtb* encounters acidic environments in the host that render it relatively resistant to all TB drugs except PZA. PZA is a key component of TB therapy but resistance to PZA is spreading. New drugs that can kill *Mtb* in an acidic environment are urgently needed. The route to their discovery can begin with a whole-cell screen for compounds that disrupt *Mtb*’s intrabacterial pH homeostasis, such as those identified here.

## Supporting Information

Figure S1 **Structures of 19 confirmed hits.**
(PDF)

Figure S2 **Fluorescent liposome assay counter screen to eliminate compounds that permeabilize lipid bilayers or function at proton carriers.** FA: fluorescein-5-(and 6-) sulfonic acid.
(PDF)

Figure S3 **Gramicidin assay to determine the ability of compounds to alter lipid bilayer material properties.**
(PDF)

Figure S4 **Comparison of 17D7 and 1048’s effects on pHIB two days after exposure to pH 4.5.** Results represent means and standard deviations for two experiments, each performed in triplicate.
(PDF)

Figure S5 **pH_IB_ of library compounds with various numbers (#) of carboxyl groups and various numbers of hydroxyl groups.** (A) Numbers of carboxyl groups in 1,980 compound library and corresponding pH_IB_. (B) Numbers of carboxyl groups of 19 hit compounds and corresponding pH_IB_. (C) Numbers of hydroxyl groups in 1,990 compound library and corresponding pH_IB_. (D) Numbers of hydroxyl groups of 19 hit compounds and corresponding pH_IB_. All pH_IB_ measurements were with 12.5 µM compound after 48 h.
(PDF)

File S1 **Supplemental information.**
(DOCX)
