# Correction: Whole Cell Screen for Inhibitors of pH Homeostasis in *Mycobacterium tuberculosis*

**DOI:** 10.1371/annotation/760b5b07-4922-42c4-b33a-162c1e9ae188

**Published:** 2013-08-20

**Authors:** Crystal M. Darby, Helgi I. Ingólfsson, Xiuju Jiang, Chun Shen, Mingna Sun, Nan Zhao, Kristin Burns, Gang Liu, Sabine Ehrt, J. David Warren, Olaf S. Andersen, Steven J. Brickner, Carl Nathan

The correct name of the eleventh author is: Olaf S. Andersen 

