# Supplementary material for: Whole Cell Screen for Inhibitors of pH Homeostasis in Mycobacterium tuberculosis
Source: PLoS One. 2013 Jul 30;8(7):e68942. doi: 10.1371/journal.pone.0068942 (PMC3728290; doi:10.1371/journal.pone.0068942)
Supplement: Figure S1 — Structures of 19 confirmed hits. (PDF) [file pone.0068942.s001.pdf]

Microbial Origin		*Plant Origin*	
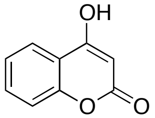 1G4	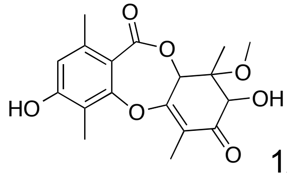 1A8	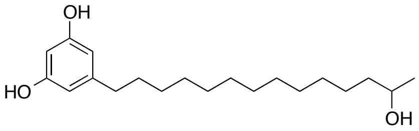 15E9	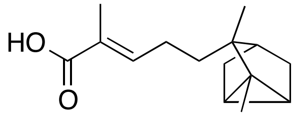 19C9
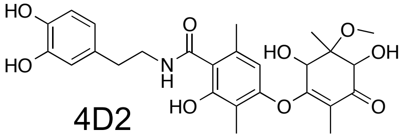 4D2	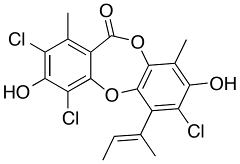 8B2 Nornidulin	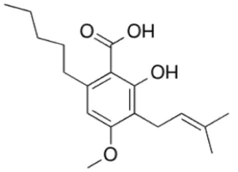 17D7	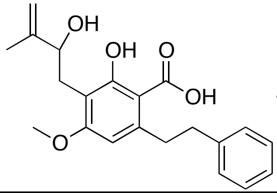 20E11
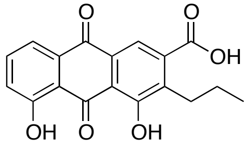 3F11	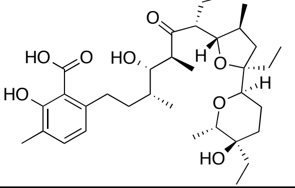 5D4 Lasalocid	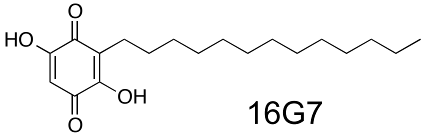 16G7 Rapanone	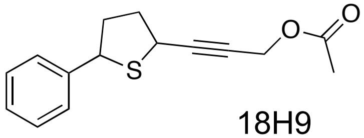 18H9
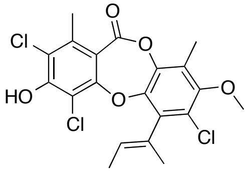 8C6 Nidulin	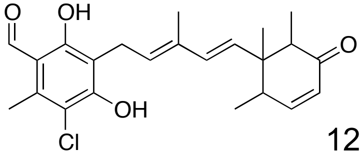 12H5		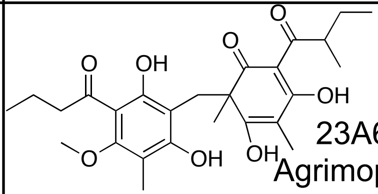 23A6 Agrimophol
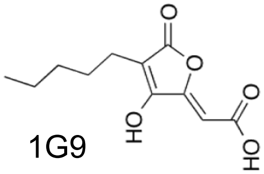 1G9	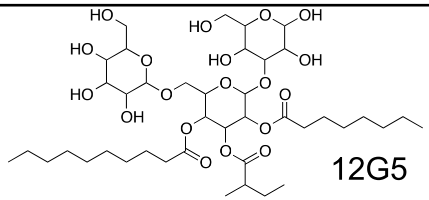 12G5		
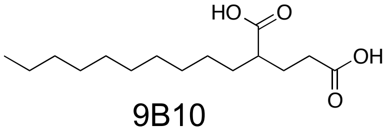 9B10	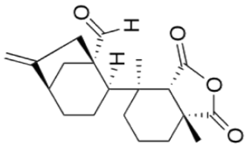 12F10		
