# Supplementary material for: Whole Cell Screen for Inhibitors of pH Homeostasis in Mycobacterium tuberculosis
Source: PLoS One. 2013 Jul 30;8(7):e68942. doi: 10.1371/journal.pone.0068942 (PMC3728290; doi:10.1371/journal.pone.0068942)
Supplement: Figure S2 — Fluorescent liposome assay counter screen to eliminate compounds that permeabilize lipid bilayers or function at proton carriers. FA: fluorescein-5-(and 6-) sulfonic acid. (PDF) [file pone.0068942.s002.pdf]

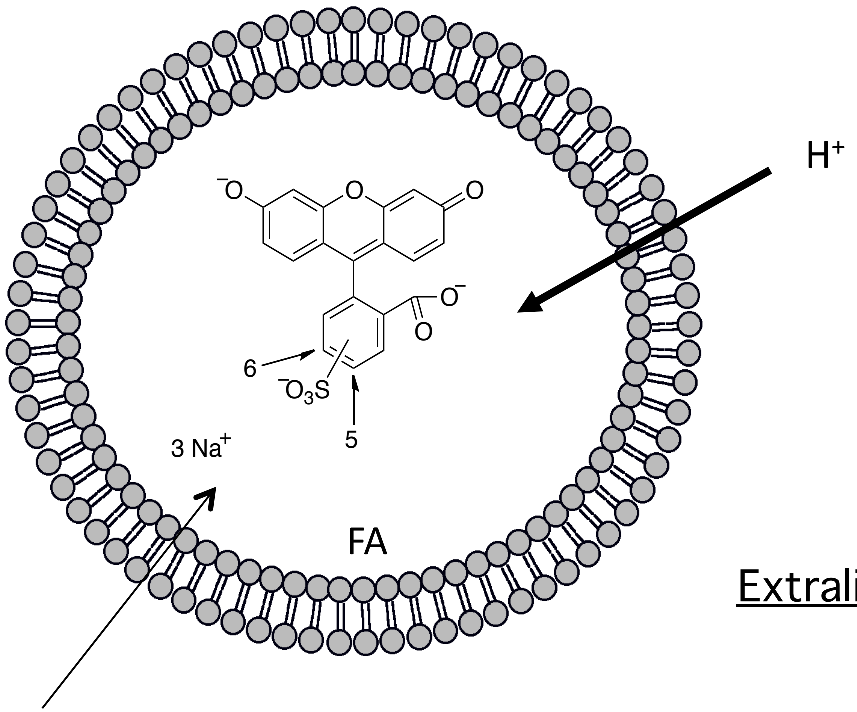

Extraliposomal Conditions

+/- compound

pH = 6.4

Intraliposomal Conditions

Fluorescein-5-(and-6)-sulfonic acid

pH = 7.4
