# Supplementary figures and images for: Whole Cell Screen for Inhibitors of pH Homeostasis in Mycobacterium tuberculosis
Source: PLoS One. 2013 Jul 30;8(7):e68942. doi: 10.1371/journal.pone.0068942 (PMC3728290; doi:10.1371/journal.pone.0068942)

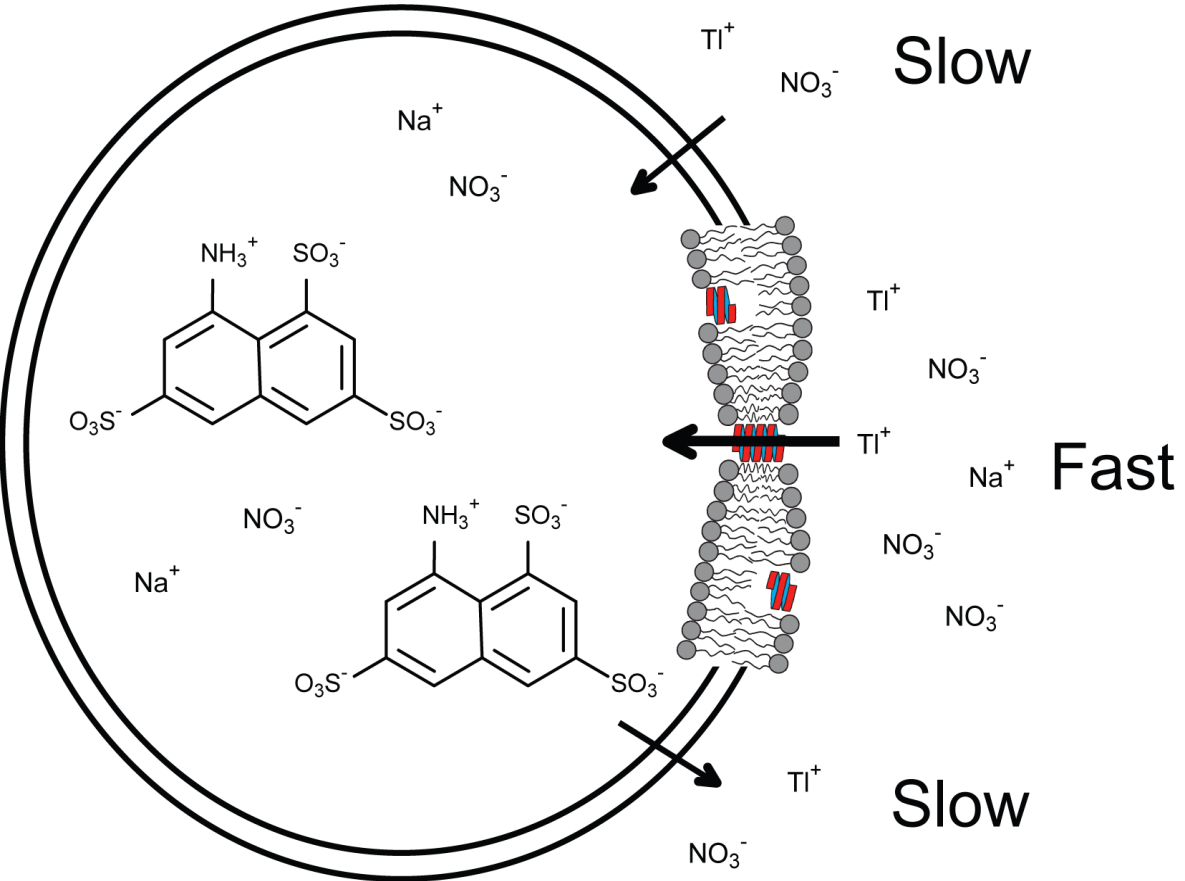

Supplement: Figure S3 — Gramicidin assay to determine the ability of compounds to alter lipid bilayer material properties. (PDF) [file pone.0068942.s003.pdf]

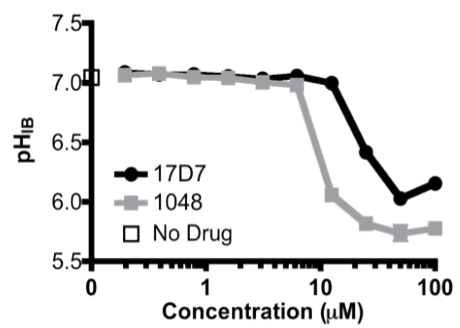

Supplement: Figure S4 — Comparison of 17D7 and 1048’s effects on pHIB two days after exposure to pH 4.5. Results represent means and standard deviations for two experiments, each performed in triplicate. (PDF) [file pone.0068942.s004.pdf]
