# Supplementary material for: Whole Cell Screen for Inhibitors of pH Homeostasis in Mycobacterium tuberculosis
Source: PLoS One. 2013 Jul 30;8(7):e68942. doi: 10.1371/journal.pone.0068942 (PMC3728290; doi:10.1371/journal.pone.0068942)
Supplement: File S1 — Supplemental information. (DOCX) [file pone.0068942.s006.docx]

***Supplemental Information for:***

**Whole cell screen for inhibitors of pH homeostasis**

**in *Mycobacterium tuberculosis***

Crystal M. Darby^1^, Helgi I. Ingólfsson^2^, Xiuju Jiang^1^, Chun Shen^3^, Mingna Sun^5^, Nan Zhao^1^, Kristin Burns^1^, Gang Liu^5,6^, Sabine Ehrt^1^, J. David Warren^3, 4^, Olaf S. Anderson^2^, Steven J. Brickner^7^, and Carl Nathan^1,8^

^1^Department of Microbiology and Immunology, ^2^Department of Physiology and Biophysics, ^3^Milstein Chemistry Core Facility and ^4^Department of Biochemistry, Weill Cornell Medical College, New York, New York, United States of America; ^5^Institute of Materia Medica, Chinese Academy of Medical Sciences & Peking Union Medical College, and ^6^Tsinghua-Peking Center for Life Sciences and Department of Pharmacology and Pharmaceutical Sciences, School of Medicine, Tsinghua University, Beijing, P. R. China; and ^7^SJ Brickner Consulting, LLC, Ledyard, Connecticut, United States of America

^8^To whom correspondence should be addressed at cnathan@med.cornell.edu

**Supplemental Experimental Procedures**

*Synthesis of compound 1048*

Unless otherwise stated, all commercially available materials were purchased from Aldrich, TCI, or Alfa Aesar and were used without any further purification. When necessary, solvents and reagents were dried prior to use, using standard protocols. All non**‐**aqueous reactions were carried out in oven**‐**dried glassware under an atmosphere of argon. Analytical thin layer chromatography (TLC) was performed on silica gel 60, F254 plates (0.25 mm thickness) from SiliCycle. Visualization was accomplished by either irradiation under a 254 nm UV lamp or by staining with an aqueous solution of ceric ammonium molybdate (CAM). Flash chromatography was performed on silica gel 60 (230**-**400 mesh). ^1^H and ^13^C NMR spectra were acquired on a Bruker DRX**‐**500 spectrometer at 500 MHz for ^1^H and 125 MHz for ^13^C. Chemical shifts are expressed in parts per million downfield from tetramethylsilane (TMS), using either TMS or the solvent resonance as an internal standard (TMS, ^1^H: 0 ppm; chloroform, ^13^C: 77.0 ppm). Data are reported as follows: chemical shift, multiplicity (s = singlet, d = doublet, t = triplet, q = quartet, m = multiplet, br = broad), coupling constant, and integration.

Methyl 2,4-dihydroxy-3-(3-methylbut-2-en-1-yl)-6-pentylbenzoate

To a well-stirred solution of methyl olivetolate ([Porwoll and Leete, 1985](#_ENREF_22)) (357 mg, 1.5 mmol) in benzene (25 mL) was added *n*-butyl lithium (0.6 mL, 2.5 M in hexane, 1.5 mmol). The mixture was stirred at ambient temperature for 1 h, at which point 1-bromo-3-methyl-2-butene (196 μL, 1.7 mmol) was added. The resulting mixture was heated at reflux for 4 h, then cooled to ambient temperature and quenched by the slow addition of ice-cold water (20 mL). The aqueous layer was adjusted to pH 4 with 2 N HCl, and then extracted with ethyl acetate (3 x 25 mL). The combined organic layers were washed with brine, dried over Na_2_SO_4_, and concentrated *in vacuo*. The title compound was obtained as a white powder (189 mg, 41%) following purification by column chromatography (10 % ethyl acetate in hexanes). ^1^H NMR (500 MHz, CDCl_3_) δ 12.01 (s, 1H), 6.23 (s, 1H), 5.78 (s, 1H), 5.35 – 5.21 (m, 1H), 3.91 (s, 3H), 3.41 (d, *J* = 7.2 Hz, 2H), 2.88 – 2.73 (m, 2H), 1.82 (s, 3H), 1.75 (s, 3H), 1.56 – 1.47 (m, 2H), 1.37 – 1.29 (m, 4H), 0.95 – 0.86 (m, 3H). ^13^C NMR (125 MHz, CDCl_3_) δ 172.6, 162.7, 159.4, 145.9, 135.3, 121.7, 111.7, 110.8, 104.8, 52.0, 36.9, 32.2, 31.7, 26.0, 22.7, 22.3, 18.0, 14.2. ESI-MS calcd for C_18_H_26_O_4_ [M+H]^+^: 307.2 found: 307.2.

2,4-dihydroxy-3-(3-methylbut-2-en-1-yl)-6-pentylbenzoic acid (1048)

To a stirred solution of lithium propyl mercaptide ([Bartlett and Johnson, 1970](#_ENREF_1)) (3 mL, 0.5 M in HMPA) at ambient temperature was added methyl 2,4-dihydroxy-3-(3-methylbut-2-en-1-yl)-6-pentylbenzoate (180 mg, 0.59 mmol). The resulting mixture was stirred for 2.5 h at ambient temperature then slowly poured into ice-cold 1 N HCl (20 mL) and extracted with diethyl ether (3 x 20 mL). The ether extract was concentrated to 15 mL and extracted with 2 N NaOH (3 x 10 mL). The combined aqueous layers were adjusted to pH 2 with 0.1 N HCl and extracted with diethyl ether (3 x 20 mL). The combined organic layers were washed with brine, dried over MgSO_4_, and concentrated *in vacuo*. The title compound was obtained as a white crystalline solid (154 mg, 90%) following purification by column chromatography (hexanes/ethyl acetate/AcOH, 10:5:0.15). ^1^H NMR (500 MHz, CDCl_3_) δ 11.88 (brs, 1H), 6.28 (s, 1H), 5.31 – 5.24 (m, 1H), 3.42 (d, *J* = 7.1 Hz, 2H), 2.93 – 2.85 (m, 2H), 1.83 (s, 3H), 1.76 (s, 3H), 1.62 – 1.53 (m, 2H), 1.39 – 1.30 (m, 4H), 0.94 – 0.87 (m, 3H). ^13^C NMR (125 MHz, CDCl_3_) δ 176.0, 163.8, 160.4, 147.6, 135.5, 121.5, 111.8, 111.3, 103.3, 36.7, 32.1, 31.6, 26.0, 22.7, 22.3, 18.1, 14.2. ESI-MS calcd for C_17_H_24_O_4_ [M+H]^+^: 293.2 found: 293.2.

**Supplemental References**

Bartlett, P.A., and Johnson, W.S. (1970). An improved reagent for the O-alkyl cleavage of methyl esters by nucleophilic displacement. Tetrahedron Letters *11*, 4459-4462.

Porwoll, J.P., and Leete, E. (1985). Synthesis of [5, 6-13C2, 1-14C] olivetolic acid, methyl [1′-13C] olivetolate and [5, 6-13C2, 1-14C] cannabigerolic acid. J Labelled Compd Rad *22*, 257-271.
