# Supplementary material for: Whole Cell Screen for Inhibitors of pH Homeostasis in Mycobacterium tuberculosis
Source: PLoS One. 2013 Jul 30;8(7):e68942. doi: 10.1371/journal.pone.0068942 (PMC3728290; doi:10.1371/journal.pone.0068942)
Supplement: Figure S5 — pHIB of library compounds with various numbers (#) of carboxyl groups and various numbers of hydroxyl groups. (A) Numbers of carboxyl groups in 1,980 compound library and corresponding pHIB. (B) Numbers of carboxyl groups of 19 hit compounds and corresponding pHIB. (C) Numbers of hydroxyl groups in 1,990 compound library and corresponding pHIB. (D) Numbers of hydroxyl groups of 19 hit compounds and corresponding pHIB. All pHIB measurements were with 12.5 µM compound after 48 h. (PDF) [file pone.0068942.s005.pdf]

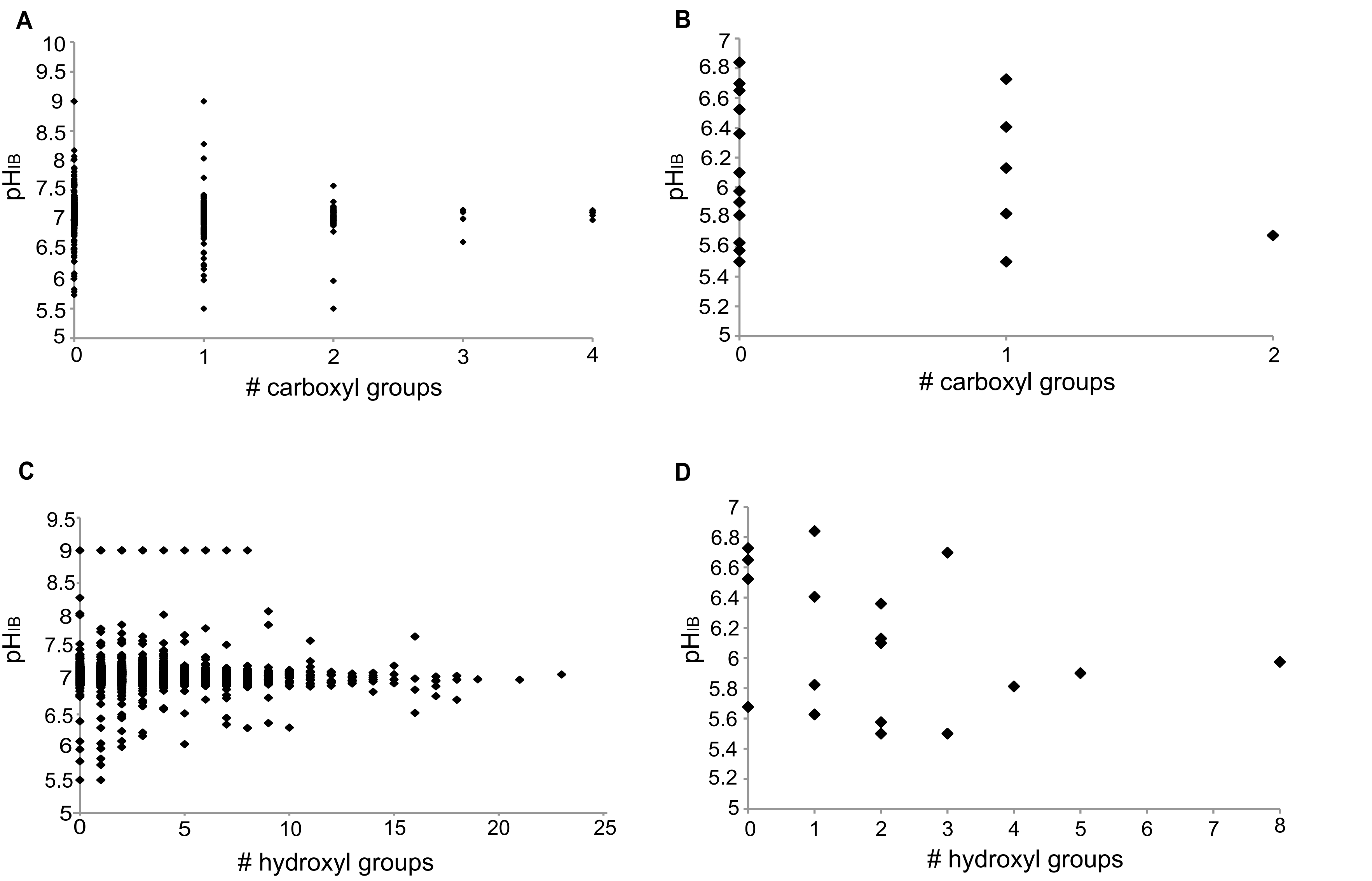

A
pHIB
10
9.5
9
8.5
8
7.5
7
6.5
6
5.5
5
0
1
2
3
4
carboxyl groups
B
pHIB
7
6.8
6.6
6.4
6.2
6
5.8
5.6
5.4
5.2
5
0
1
2
carboxyl groups
C
pHIB
9.5
9
8.5
8
7.5
7
6.5
6
5.5
5
0
5
10
15
20
25
hydroxyl groups
D
pHIB
7
6.8
6.6
6.4
6.2
6
5.8
5.6
5.4
5.2
5
0
1
2
3
4
5
6
7
8
hydroxyl groups
